# Whole-Genome Bisulfite Sequencing (WGBS) Analysis of *Gossypium hirsutum* under High-Temperature Stress Conditions

**DOI:** 10.3390/genes15101241

**Published:** 2024-09-24

**Authors:** Zhaolong Gong, Juyun Zheng, Ni Yang, Xueyuan Li, Shuaishuai Qian, Fenglei Sun, Shiwei Geng, Yajun Liang, Junduo Wang

**Affiliations:** Cash Crops Research Institute, Xinjiang Academy of Agricultural Sciences, Urumqi 830091, China; g15981775091@163.com (Z.G.); zjypp8866@126.com (J.Z.); yangni157@163.com (N.Y.); xjmh2338@163.com (X.L.); 17509977905@163.com (S.Q.); xjsunfenglei@163.com (F.S.); gengshiwei20201231@163.com (S.G.); 13579975299@163.com (J.W.)

**Keywords:** *G. hirsutum*, high temperature, WGBS, RNA-seq

## Abstract

Background: DNA methylation is an important part of epigenetic regulation and plays an important role in the response of plants to adverse stress. Methods: In this study, whole-genome bisulfite sequencing (WGBS) was performed on the high-temperature-resistant material Xinluzao 36 and the high-temperature-sensitive material Che 61–72 at 0 h and 12 h under high-temperature stress conditions. Results: The results revealed that the *Gossypium hirsutum* methylation levels of CG and CHG (H = A, C, or T) decreased after the high-temperature stress treatment, and the methylation level of the A subgenome was significantly greater than that of the D subgenome. The methylation level of CHH increased, and the methylation level of CHH in the D subgenome was significantly greater than that in the A subgenome after high-temperature stress treatment. The methylation density of CG is lower than that of CHG and CHH, and the methylation density of the middle region of chromosomes is greater than that of both ends, which is opposite to the distribution density of genes. There were 124 common differentially methylated genes in the CG, CHG, and CHH groups, and 5130 common DEGs and differentially methylated genes were found via joint analysis with RNA-seq; these genes were significantly enriched in the biosynthesis of plant hormones, thiamine metabolism, glutathione metabolism, and tyrosine metabolism pathways. DNA methylation did not affect the expression of many genes (accounting for 85.68% of the differentially methylated genes), DNA methylation-promoted gene expression was located mainly in the downstream region of the gene or gene body, and the expression of inhibitory genes was located mainly in the upstream region of the gene. Conclusions: This study provides a theoretical basis for further exploration of the gene expression and functional regulatory mechanism of *G. hirsutum* DNA methylation under high-temperature stress conditions.

## 1. Introduction

Cotton, an important fiber and oil crop, occupies an important position in the world economy [1]. *Gossypium hirsutum*, which is native to Central America, is the world’s most widely cultivated cotton and has the greatest production. Global warming has increased high-temperature stress conditions, significantly impacting global crop yields [2]. It is estimated that for every 1 °C increase in the global average temperature, wheat, rice, corn, and soybean yields may decline by approximately 6%, 3.2%, 7.4%, and 3.1%, respectively [3]. It is estimated that by 2050, global warming may reduce crop yields by one-third [4]. However, the high-temperature weather in Xinjiang, China, from July to August is highly synchronized with the cotton flowering and boll period, which results in a decrease in pollen vitality and the drying and falling of young cotton buds. A significant reduction in boll formation in the upper part (inverted third fruit branch) results in a large number of buds and bolls falling off. Additionally, reduced yield and deterioration of quality occur, which has become an important problem restricting cotton production [5,6]. High temperatures can also cause cotton to bloom early, which shortens its growth period, resulting in a decrease in fiber yield and quality [7]. The cultivation of new varieties of high-temperature-resistant cotton will be an important technique to cope with future high temperatures and break the bottleneck of perennial yield lingering.

In recent years, with the increase in global warming and the resulting increase in severe high-temperature stress conditions, there has been a growing focus on the mechanisms behind crop resilience to high temperatures. DNA methylation is the process whereby specific bases in the DNA sequence acquire a methyl group through covalent bonding catalyzed by DNA methyltransferase. This modification primarily occurs in CpG dinucleotides [8]. During cytosine methylation, the fifth carbon atom is modified to produce 5-methylcytosine (5 mC), which is the predominant form of DNA methylation in eukaryotic organisms, including plants and animals [9]. Studies have indicated that, under adverse conditions, the DNA methylation levels in plants can either significantly increase or decrease compared with those observed under normal, non-adverse conditions [10]. This finding shows that plants control the expression of certain genes through their own DNA methylation to better adapt to adversity [10]. Young leaves from rice plants subjected to high-temperature stress treatment were collected at seven time points for methylation-sensitive amplification polymorphism analysis. The high-temperature stress treatment resulted in a reduction in the overall methylation levels of the rice, with two associated genes showing a negative correlation with methylation levels. By modulating the expression levels of these genes, the resistance of rice to high temperatures can be modified [11]. Studies have demonstrated that the fertility of cotton anthers can be restored under high-temperature stress conditions. Treatment with methylation inhibitors during high-temperature stress triggers DNA demethylation in cotton plants, facilitating the normal development of cotton anthers [12]. The use of methylation promoters in cotton under high-temperature stress conditions led to an increase in the DNA methylation levels of cotton, consequently impeding the development of certain anthers [12]. Following high-temperature stress treatment, cucumber seedlings presented elevated leaf methylation levels, in contrast with the ongoing decrease in overall methylation levels observed in young leaves of four-leafed rice seedlings as high-temperature stress progresses [13]. During high-temperature stress treatment, the methylation level decreases or increases, indicating that temperature stress can induce plants to undergo methylation or demethylation to better adapt to adverse conditions [14].

In the past, research on high-temperature stress in cotton has focused mainly on physiology, biochemistry, and expression regulation [15,16,17]. In recent years, with the continuous advancements in genetics and molecular biology technology research, studies have revealed that epigenetics plays an important role in the resistance of model plants such as tobacco, Arabidopsis, and rice to environmental stress. As an important component of epigenetic research, DNA methylation is involved in the genome defense of organisms, gene expression regulation, and the growth and development of plants, as well as the whole process of resistance to adversity [18,19]. At present, the study of DNA methylation has become an important aspect of epigenetic research, and many studies have been carried out on the regulatory mechanism and role of DNA methylation in model plants. With the continuous deepening of DNA methylation research, analysis of the mechanism of DNA methylation and the relationship between methylation and gene expression will provide some ideas for the molecular breeding of animals and plants. Tolerance to environmental temperature stress is an important trait in cotton breeding and is controlled by genetic inheritance and epigenetic regulation of DNA and histone modifications [20,21]. A previous study revealed that changes in histone modifications (H3K4me3 and H3K9ac) were positively correlated with the activation of cotton cold stress response genes [22]. Compared with those under normal conditions, CG, CHG (H = A, C, or T), and CHH under drought stress and rewatering conditions presented hypermethylation patterns and returned to normal levels after rewatering [23]. Although the relationship between methylation and high-temperature resistance has been reported in cotton, the mechanism by which DNA methylation regulates high-temperature resistance in cotton and the regulatory relationship with gene expression patterns still need further in-depth research. This study investigated the changes in the methylation (5 mC) level of the whole-genome DNA of the heat-resistant material Xinluzao 36 and the heat-sensitive material Che 61–72 at different time points under high-temperature stress conditions to analyze the methylation levels of CG, CHG, and CHH in the two materials before and after stress, as well as the methylation levels between the subgenomes. Through the use of methylation combined with RNA-seq, understanding how DNA methylation regulates the expression of stress-related genes and stress-related metabolic pathways in upland cotton under high-temperature stress conditions at the transcriptional level is possible. These findings provide a theoretical basis for the development of new methods for studying the molecular breeding of cotton for high-temperature resistance, thereby further understanding the mechanism of high-temperature resistance in cotton.

## 2. Materials and Methods

### 2.1. Plant Materials

In this study, a high-temperature resistant material, Xinluzao 36, and a high-temperature sensitive material, Che 61–72, were selected. After the experimental material seeds were sterilized in a 15% hydrogen peroxide solution, they were rinsed 2 times with sterile water. The seeds were transferred to germination bins (12 cm × 12 cm × 6 cm) filled with sterilized sand, and 10 seeds were sown 2 cm deep at equal intervals in each germination box. After sowing, 300 mL of deionized water was added to the tank to saturate the sandy soil, 200 mL of water was added every 2 days, and the germination box was placed in an illuminated incubator. The growth conditions were as follows: 16 h of light at 28 °C and 8 h of darkness at 20 °C, an optical density of 300 μmol m^−2^ s^−1^, and a relative humidity of 75%. When the cotton seedlings had grown to the three-leaf stage, the leaf samples were collected at 0 h and 12 h under high-temperature stress conditions at 40 °C in a light incubator, and each sample was subjected to 3 biological replicates, frozen in liquid nitrogen for 2 h, and then transferred to a −80 °C freezer for storage.

### 2.2. DNA Methylation Library Sequencing

After the genomic DNA sample was quantified, it was fragmented via ultrasonication and subsequently purified. The fragmented DNA underwent end repair, “A” was added to the 3′ end, and a methylated linker was introduced. The fragment size was determined via agarose gel electrophoresis. The DNA was subsequently treated with bisulfite, purified, and recovered for PCR amplification. The amplified libraries were purified and quarantined, and the qualified libraries were sequenced via the Illumina platform. The raw sequencing data were qualitatively controlled via fastp software, which removed adaptors, low-quality reads, and reads containing too many Ns [24]. BSMAP software was used to compare the filtered clean reads to the analysis of the *G. hirsutum* (https://www.CottonGen.org/species/Gossypium_hirsutum/ZJU-AD1_v2.1 (accessed on 15 March 2023)) reference genome [25,26]. BSMAP combines an algorithm of genomic hash tables and bitwise masking to obtain fast and accurate sequence alignment for bisulfite conversion. It does not convert the genome sequence but allows the Cs and Ts in the sequence to be aligned to the Cs in the genome to match the transformed sequence to the normal sequence of the genome.

### 2.3. DNA Methylation Data Analysis

MethylDackel was used for methylation site extraction. Methylation sites were identified by aligning the C sites on the reference genome with the corresponding bases [27]. Methylation was determined by the presence of a C base in the alignment. A binomial distribution test (B(n,p)) was conducted for each C site via the results of Bismark’s methylation assay to confirm genuine methylation sites [28]. To validate the reliability of detecting × methylated C sites at a specific location given a read coverage of n and a methylation non-conversion rate of p, the probability of observing × methylated C sites under these conditions needs to be assessed. Methylation sites were considered significant if the sequencing depth was 5 or greater and if the q value was 0.05 or lower. The methylation level (ML) was calculated as mC/(mC + umC), where mC represents methylated Cs and umC denotes unmethylated Cs. This method determines the distribution of methylation levels at each C site within different sequence contexts.

### 2.4. RNA-seq

The RNA extraction and transcriptome sequencing of the samples were completed by Beijing Biomec Biotechnology Co., Ltd., Beijing, China. Full-length sequences were aligned to the reference genome via Minimaxap2 software to acquire non-redundant transcript sequences. Gene expression levels were quantified by the number of genes per 10,000 reads [29]. DESeq2 (version 1.44.0) was used for differential gene screening, with a fold change ≥ 2 and an FDR < 0.01 [30]. The GOseq R software package (version 1.56.0) and KOBAS software (version 3.0) were used to determine the enrichment of GO and KEGG functions, respectively [31,32]. The expression values of the DEGs screened above were subjected to WGCNA via the R package [33]. The module was generated via the automatic network-building function with default settings. The Pearson correlation coefficient was applied to evaluate the correlation between each module and sample. A weighted network diagram was subsequently created via the OmicShare tool (http://www.omicshare.com/tools, (accessed on 22 June 2023).

### 2.5. Combined Methylation and RNA-seq Analysis

DMR (differentially methylated region) analysis was performed via Metilene software [34]. The software uses a binary segmentation algorithm to classify a certain range of C sites into a region to be detected according to the distance between C sites and then combines two statistical tests (the MWU test and 2D KS test) to quickly realize DMR redetection between pairs of samples or between two groups of samples. Finally, the differentially methylated region was obtained via multiple test corrections. The CG, CHG, and CHH sites were used to look for differentially methylated regions. KEGG enrichment analysis was performed for the differentially methylated genes and DEGs via the clusterProfiler software package (version: 4.12.6).

## 3. Results

### 3.1. Effects of High Temperature on the Methylation of G. hirsutum

**Figure 1:**
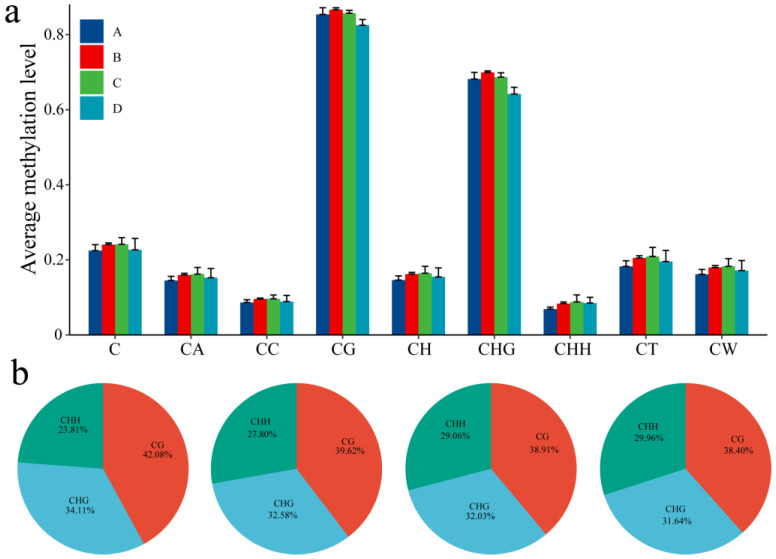
(**a**) Cytosine and average methylation levels of each context at 0 h and 40 h under the high-temperature stress treatment for Xinluzao 36 and Che 61–72. (**b**) The proportion of methylated cytosine in Xinluzao 36 and Che 61–72 under the high-temperature stress treatment at 0 h and 40 h.

To explore the high-temperature resistant material Xinlu Zao 36 and the high-temperature sensitive material Che 61–72, they were subjected to high-temperature stress treatment for 0 h and 12 h via whole-genome bisulfite sequencing (WGBS). The clean data of 1022.17 Gb were collected after filtration, and the sequencing depth was greater than 30×. The Q20 percentage was ≥98.22%, the Q30 percentage was ≥95.14%, the GC content was ≥21.65%, the alignment rate with the reference genome was 80.25~83.38%, and the average alignment rate was 81.89% (Table S1). These results indicate that the sequencing data were acceptable for subsequent analysis. First, the average methylation level of cytosine and each context was determined, and the average methylation level of CG was greater than 0.8 in the four samples. The average methylation level of CHG (H represents bases other than guanine) was approximately 0.7. The remaining genes (including C, CA, CC, CH, CHH, CT, and CW) presented mean methylation levels of less than 0.3 (Figure 1a). Among the Xinlu Zao 36 samples subjected to high-temperature stress treatment for 0 h, the proportion of CG methylation was the highest, at 42.08%, the proportion of CHG was 34.11%, and the proportion of CHH was 23.81% (Figure 1b). Xinlu Zao 36 was treated at a high temperature for 12 h, and the proportion of CG methylation was the highest, at 36.92%, the proportion of CHG was 32.58%, and the proportion of CHH was 27.80%. Under the high-temperature stress treatment of 61–72 for 0 h, the proportion of CG methylation was the highest, at 38.91%, the proportion of CHG was 32.03%, and the proportion of CHH was 29.06% (Figure 1b). After 12 h of high-temperature stress treatment with car 61–72, the proportion of CG methylation was the highest, at 38.40%, the proportion of CHG was 31.64%, and the proportion of CHH was 29.96%. These results reveal that the proportion of methylated cytosine in CG and CHG decreased after the high-temperature stress treatment, and the decrease was less pronounced in the high-temperature-resistant materials (CG and CHG decreased by 0.51% and 0.39%, respectively), the methylation level of CHH increased, and the review level of CHH decreased (0.90%) in the high-temperature-resistant materials.

**Figure 2:**
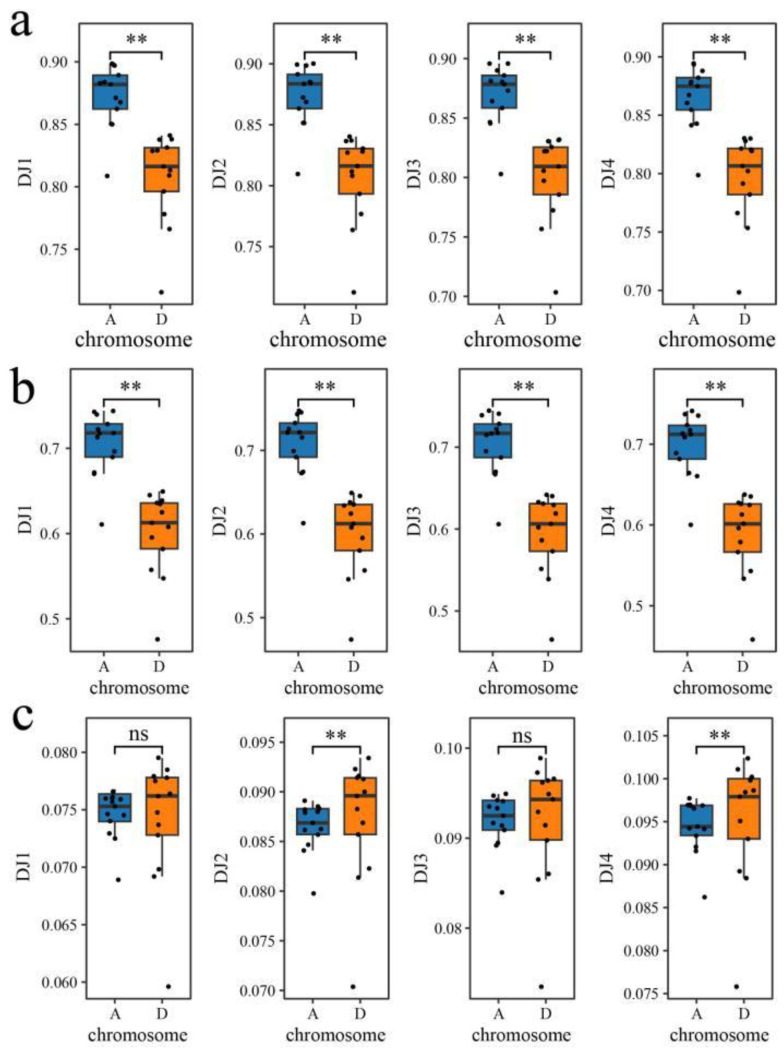
(**a**) CG methylation levels of each subgenome at 0 h and 40 h under the high-temperature stress treatment in Xinluzao 36 and Che 61–72; (**b**) CHG methylation levels in each subgenome at 0 h and 40 h under the high-temperature stress treatment in Xinluzao 36 and Che 61–72; and (**c**) CHH methylation levels in each subgenome at 0 h and 40 h under the high-temperature stress treatment in Xinluzao 36 and Che 61–72. (error bars represent the mean ± SE of three replicates, ** *p* < 0.01. ns means no statistical significance).

Comparative analysis of the methylation levels of C sites in different subgenomes revealed that the methylation levels of CG and CHG in *G. hirsutum* subgenome A were significantly greater than those in the D subgenome (Figure 2). There was no significant difference in the methylation level of CHH between the A and D subgenomes before the high-temperature stress treatment, the methylated water level of the D subgenome was significantly greater than that of the A subgenome after the high-temperature stress treatment, the methylation level of CHH was greater than that of the sensitive and high-temperature materials in the high-temperature-resistant materials, and the methylation level of CHH was increased by the high-temperature stress treatment.

### 3.2. Analysis of Dynamic Changes in DNA Methylation Levels

**Figure 3:**
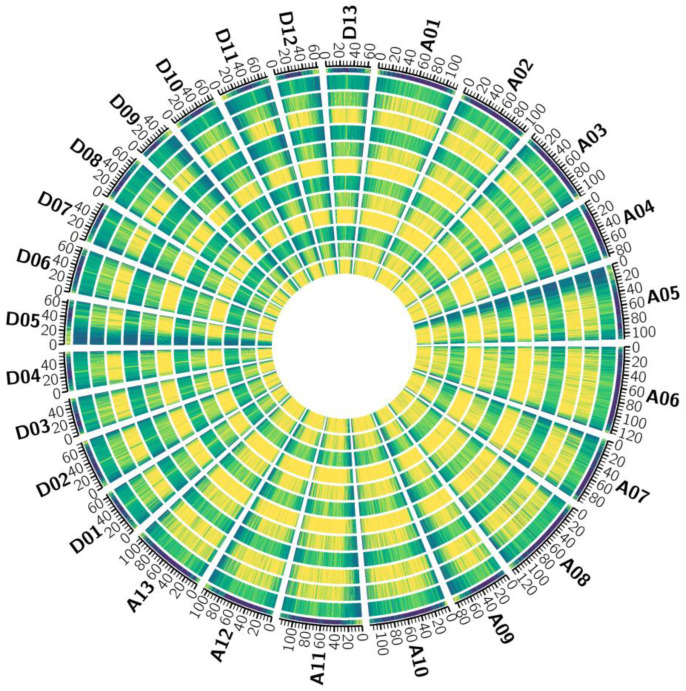
The methylation distribution map from outside to inside was as follows: gene density, CG (car 61–72, 0 h), CHG (car 61–72, 0 h), CHH (car 61–72, 0 h), CG (car 61–72, 12 h), CHG (car 61–72, 12 h), CHH (car 61–72, 12 h), CG (Xinluzao 36, 0 h), CHG (Xinluzao 36, 0 h), CHH (Xinluzao 36, 0 h), CG (Xinluzao 36, 12 h), CHG (Xinluzao 36, 12 h), and CHH (Xinluzao 36, 12 h) chromosomal range density distribution information. The blue-to-yellow color represents the direction in which the density increased.

The changes in the genome methylation level in the cotton leaves after the high-temperature stress treatment were further analyzed, the methylation level of each window at a size of 100 K was calculated, and the C-base methylation level of different samples was analyzed at the chromosome level (Figure 3). The CG methylation density of each chromosome was lower than that of CHG and CHH at the four treatment periods for the two materials, and the methylation density of the middle region of each chromosome was greater than that of both ends, which was opposite to the distribution density of genes. The methylation density of CHG and CHH in the A subgenome was greater than that in the D subgenome. On each chromosome of the D subgenome, the methylation density of CHH was greater than that of CG and CHG.

**Figure 4:**
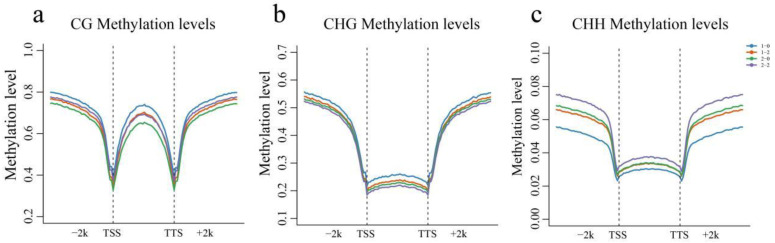
(**a**) CG methylation levels in genetically different regions of the genome (upstream, gene body, and downstream); (**b**) CHG methylation levels in genetically different regions of the genome (upstream, gene body, and downstream); and (**c**) CHH methylation levels in genetically different regions of the genome (upstream, gene body, and downstream).

According to the location of the methylation sites on the reference genome and the gene location information on the reference genome, the genomic regions (upstream (2 kb), gene body, and downstream (2 kb)) were divided into 40 bins to count the methylation levels of different regions of the whole genome (Figure 4). In terms of the methylation level 2 kb upstream and downstream of the gene, the methylation level of the CG type increased with increasing distance from the gene, and the methylation level in the middle region of the gene was greater than that at both ends (Figure 4a). The methylation level of the CG type was greater than that of Che 61–72 (high-temperature sensitive) in Xinluzao 36 (high-temperature resistant). The methylation level in the upstream and downstream 2 kb regions of the gene decreased after the high-temperature stress treatment, the CG methylation level of the high-temperature-resistant materials decreased in the gene region, and the methylation level of the sensitive high-temperature materials increased. The methylation level of the CHG type increased with increasing distance from the gene, and the methylation level in the gene region changed only slightly (Figure 4b). The methylation level of the CHG type was greater than that of the sensitive high-temperature materials in the high-temperature-resistant materials, and the methylation levels of the gene body and upstream and downstream 2 kb genes decreased after the high-temperature stress treatment. The methylation level of the CHH type decreased further from the gene, and the methylation level in the gene region changed only slightly (Figure 4c). The methylation level of the CHH type was lower than that of the sensitive high-temperature materials in the high-temperature-resistant materials, and the methylation level of the gene body and upstream and downstream 2 kb increased after the high-temperature stress treatment.

### 3.3. Differentially Methylated Region (DMR) Analysis

**Figure 5:**
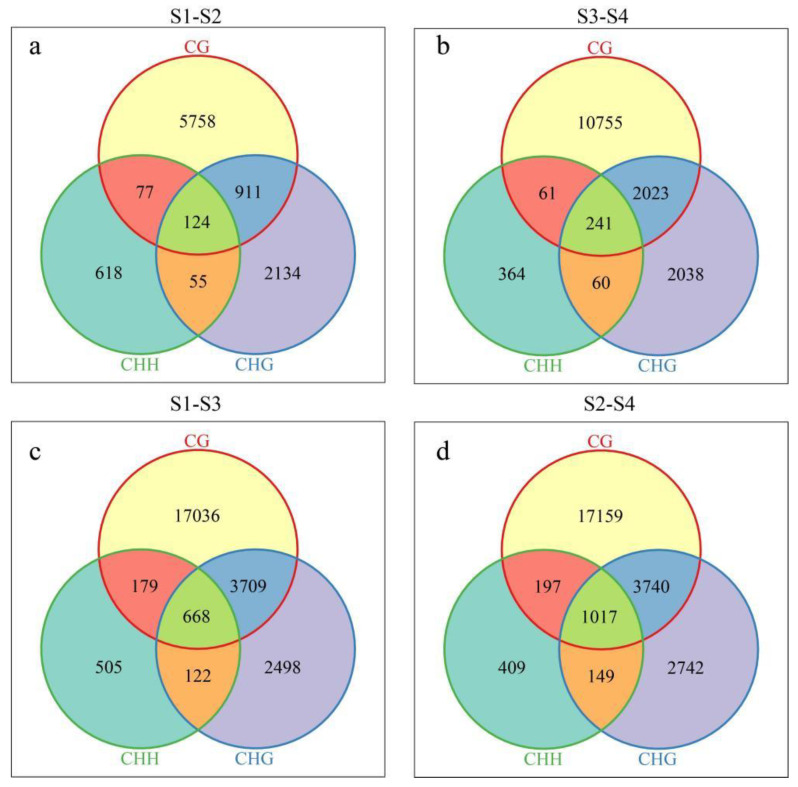
(**a**) Venn diagram of the differential methylation regions (DMRs) at 0 h and 12 h under high-temperature stress treatment; different colors represent different methylation types, and the numbers represent the number of DMRs. (**b**) Venn diagram of the differential methylation regions (DMRs) at 0 h and 12 h under high-temperature stress treatment; the different colors represent different methylation types and the numbers represent the number of DMRs. (**c**) Venn diagram of the differential methylation regions (DMRs) at 0 h under high-temperature stress treatment; the different colors represent different methylation types and the numbers represent the number of DMRs. (**d**) Venn diagram of the differential methylation regions (DMRs) at 6 h and 72 h under high-temperature stress treatment; the different colors represent different methylation types and the numbers represent the number of DMRs.

A Venn map was generated for the DMR-anchored genes and promoter region-related genes via differential methylation analysis of the CG, CHG, and CHH sequences (Figure 5). There were 124 common differentially methylated genes for the CG, CHG, and CHH types; 5758 unique differentially methylated genes for the CG type; 2134 unique differentially methylated genes for the CHG type; and 618 unique differentially methylated genes for the CHH type (Figure 5a) under the high-temperature stress treatment for Xinlu Zao 36. There were 241 common differentially methylated genes (CBs) in the CG, CHG, and CHH types; 10,755 unique differentially methylated genes in the CG type; 2038 unique differentially methylated genes in the CHG type; and 364 unique differentially methylated genes (Figure 5b) in the CHH type between 0 h and 12 h of the high-temperature stress treatment for Che 61–72. There were 668 common differentially methylated genes in the CG, CHG, and CHH types; 17,036 unique differentially methylated genes in the CG type; 2498 unique differentially methylated genes in the CHG type; and 505 unique differentially methylated genes in the CHH type (Figure 5c) between Xinlu Zao 36 and Che 61–72 under the high-temperature stress treatment. There were 1017 common differentially methylated genes for the CG, CHG, and CHH types; 17,159 unique differentially methylated genes for the CG type; 2742 unique differentially methylated genes for the CHG type; and 409 unique differentially methylated genes for the CHH type (Figure 5c) after 12 h of the high-temperature stress treatment for Xinluzao 36 and Che 61–72.

### 3.4. RNA-seq Combined with Methylation

**Figure 6:**
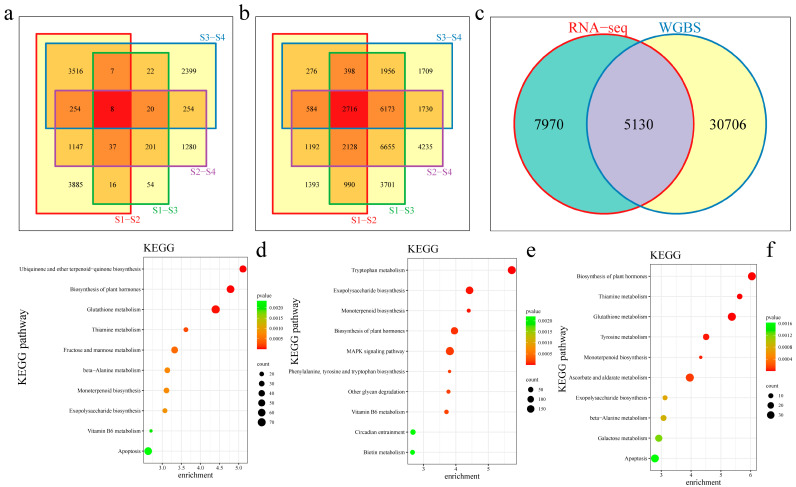
(**a**) Venn diagram of the differentially expressed genes identified via RNA-seq; (**b**) Venn diagram of the differentially methylated genes; (**c**) Venn diagram of the differentially methylated genes and the DEGs; (**d**) scatter plot of the DEGs identified via KEGG enrichment analysis; (**e**) scatter plot of the differentially methylated gene KEGG enrichment analysis; and (**f**) KEGG enrichment analysis scatter plot of the differentially methylated genes and common DEGs.

To further study the association between DNA methylation and gene expression in high-temperature-stress-treated *G. hirsutum*, we performed differential expression analysis on RNA-seq data from the same sample (Figure 6a). There were 13,100 DEGs before and after the high-temperature stress treatment of Xinlu Zao 36 and Che 61–72, including eight common DEGs. There were 3885 unique DEGs between 0 h and 12 h in Shinriku Zao 36, 54 unique DEGs between 0 h in Shinriku Zao 36 and Che 61–72, 1280 unique DEGs between 12 h in Shinriku Zao 36 and Che 61–72, and 2399 unique DEGs between 0 h and 12 h in Che 61–72. A total of 35,836 differentially methylated genes were found in Xinluzao 36 and Che 61–72 before and after the high-temperature stress treatment, including eight common differentially methylated genes (Figure 6b). There were 1393 unique differentially methylated genes between 0 h and 12 h in Xinluzao 36, 3701 unique differentially methylated genes between 0 h in Xinlu Zao 36 and Che 61–72, 4235 unique differentially methylated genes between 12 h in Xinlu Zao 36 and Che 61–72, and 1709 unique differentially methylated genes between 0 h and 12 h in Che 61–72. There were 5130 common DEGs and differentially methylated genes, 7970 unique DEGs, and 30,706 unique differentially methylated genes (Figure 6c). These results indicate that DNA methylation did not affect the expression of many genes (accounting for 85.68% of the differentially methylated genes) under the high-temperature stress treatment. Through joint analysis of the transcriptome and methylation data, a systematic discussion of the relationships between the DEGs in *G. hirsutum* and the differentially methylated genes under the high-temperature stress treatment was conducted. KEGG pathway enrichment analysis revealed that 13,100 DEGs were significantly enriched in KEGG pathways such as ubiquinone and other terpenoid-quinone biosynthesis, the biosynthesis of plant hormones, and glutathione metabolism (Figure 6d). A total of 35,836 differentially methylated genes were significantly enriched in tryptophan metabolism, exopolysaccharide biosynthesis, monoterpenoid biosynthesis, and the biosynthesis of plant hormone pathways (Figure 6e). A total of 5130 common DEGs and differentially methylated genes were significantly enriched in the following KEGG pathways: the biosynthesis of plant hormones, thiamine metabolism, glutathione metabolism, and tyrosine metabolism (Figure 6f).

### 3.5. WGCNA

**Figure 7:**
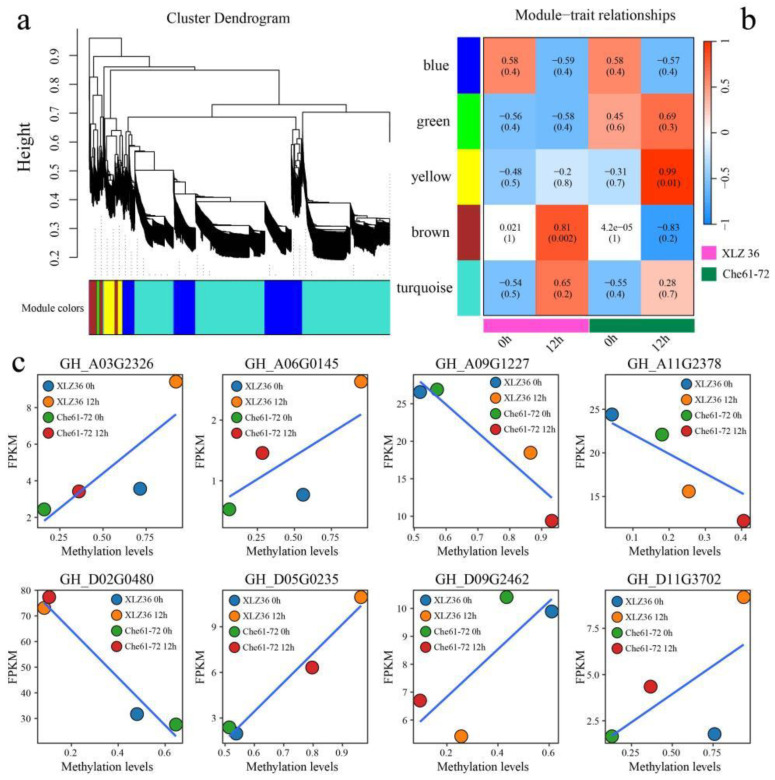
(**a**) WGCNA gene clustering dendrogram; (**b**) correlation and significance of the module with Xinluzao 36 and Che 61–72 at 0 h and 12 h; and (**c**) linear relationship between the expression levels of eight candidate genes and methylated water.

Based on the expression levels of 5130 common DEGs and differentially methylated genes, β = 4 was selected to construct the network, the dynamic shear tree method was used to merge the similarly expressed modules, and a total of five coexpression modules (Figure 7a) were obtained. The yellow module was significantly correlated with the high-temperature stress treatment of Che 61–72 after 12 h, and the brown module was significantly correlated with the high-temperature stress treatment of Xinlu Zao 36 after 12 h (Figure 7b). Each module identified the four genes with the highest connectivity as hub genes and ultimately obtained eight hub genes (Figure S1). The relationships between the expression levels of these eight candidate genes and methylated water were further calculated, and the expression levels of five genes (*GH_A03G2326*, *GH_A06G0145*, *GH_D05G0235*, *GH_D09G2462*, and *GH_D11G3702*) were positively correlated with the methylation level, whereas those of three genes (*GH_A09G1227*, *GH_A11G2378*, and *GH_D02G0480*) were inversely correlated with the methylation level. Methylation of GH_A03G2326 occurs in the gene body region, and methylation of *GH_A06G0145*, *GH_D05G0235*, *GH_D09G2462*, and *GH_D11G3702* occurs in the 2 kb region downstream of the gene body. *GH_A09G1227*, which involves the methylation of *GH_A11G2378* and *GH_D02G0480*, is located in the 2 kb region upstream of the gene body. These findings indicate that *G. hirsutum* DNA methylation can promote or inhibit gene expression at the transcriptional level under high-temperature stress conditions and that the methylation that promotes gene expression is located mainly in the downstream or gene region, whereas the methylation that inhibits gene expression is located mainly in the upstream region of the gene.

## 4. Discussion

DNA methylation is one of the clearest mechanisms in epigenetics research; it is widely used in the biological world and is the most common genome modification method and regulates genome function without changing the base sequence of DNA [35]. The DNA methylation level can affect plant growth and development, change the flowering period of plants, help plants resist adverse stress, effectively regulate genes, and ensure genome stability [36,37,38]. In the face of adverse conditions in plants, epigenetic variation changes the conformation of plant DNA, thereby changing the structure of chromatin and protein and regulating the genome [36,37,38]. As the temperature changes, plants maintain their stability through reversible dynamics of DNA methylation and demethylation. In this study, whole-genome methylation sequencing (WGBS) was performed on the genomes of the *G. hirsutum* high-temperature-resistant material Xinluzao 36 and the high-temperature-sensitive material Che 61–72 after high-temperature stress treatment for 0 h and 12 h. After filtering, a total of 1022.17 Gb of clean data was obtained, and the sequencing depth exceeded 30×. Some studies have shown that the DNA methylation level of American ginseng Lutou increased under low-temperature conditions, decreased at the beginning of spring, and increased during the whole seedling stage from early spring to late autumn [39]. At low temperatures, the DNA demethylase *DML2* is inhibited, resulting in excessive DNA methylation, which significantly delays fruit ripening and the formation of flavor volatiles, which explains why the flavor quality of ripe tomato fruits is greatly reduced after refrigeration [40]. Overall, plants exposed to high temperatures generally exhibit lower methylation levels than control plants, including *Populus simonii* and rapeseed [41,42]. High-temperature stress influences cytosine methylation in various genes. Compared with heat-resistant genotypes, heat-sensitive genotypes typically present higher methylation levels under high-temperature stress conditions. Heat-resistant genotypes exhibit more DNA demethylation events, while heat-sensitive genotypes present more DNA methylation events [43]. When we subjected cotton to high-temperature stress, we found that genomic DNA methylation and demethylation occurred simultaneously, but there were obvious material differences; methylation occurred mainly in the high-temperature-resistant Xinluzao 36, and demethylation occurred in the high-temperature-sensitive Che 61–72. We also determined the average methylation levels of cytosine and each context in the genomes of Xinluzao 36 and Che 61–72 after 0 h and 12 h of high-temperature stress treatment and reported that the proportion of methylated cytosine in CG and CHG decreased after the high-temperature stress treatment and decreased less in the high-temperature-resistant materials (CG and CHG decreased by 0.51% and 0.39%, respectively). These results suggest that cotton can maintain genome stability through DNA methylation changes, thereby resisting high-temperature stress, and that the DNA methylation level and state are related to the heat tolerance of varieties. During high-temperature stress in cotton anthers, genomic DNA methylation undergoes changes. The high-temperature-tolerant line presented significant hyper-CHH methylation, whereas the high-temperature-sensitive line presented low CHH methylation during the tetrad and layer degradation stages. These observations align with the patterns observed in our results at the seedling stage [44].

Gene expression in peach fruit is significantly associated with the methylation level of the promoter region. A negative correlation exists between the methylation level of the promoter and gene expression levels in peach fruit. Elevated methylation levels in the promoter region of peach fruit result in the downregulation of gene expression [45]. In pear fruits, methylation in specific regions (−604 to −911 bp and −1218 to −1649 bp) of the *MYB10* promoter suppresses MYB10 expression, subsequently inhibiting anthocyanin synthesis in the peel [46]. An examination of the methylation levels across various regions of peach peel genes revealed predominant CG methylation levels in peach fruits. Following a 3-day storage period, a notable increase in methylation levels was observed in the promoter region, as well as in the gene body and the regions 2 kb upstream and downstream of the gene [47]. As a result, there is an increase in the degree of methylation during natural storage [48]. The DNA methylation level of citrus fruits increases during storage, and the ripening of citrus fruits is closely related to the methylation level [49]. The increase in DNA methylation and decrease in flavor compounds in peach fruit due to low-temperature stress are considered mechanisms of chilling injury. Treatment with methyl jasmonate alleviated chilling injury by inducing significant alterations in gene expression and promoter methylation status [45]. These results indicate that during storage, plant hormones affect the DNA methylation level of the fruit, thereby regulating the transcription level. We identified 35,836 differentially methylated genes from WGBS data analysis before and after the high-temperature stress treatment of cotton, accounting for 49.25% of the total number of *G. hirsutum* genes. The methylation region of the promoter region of the 11,693 genes is similar to the mechanism of action of nitric oxide, which involves the mediation of DNA methylation to increase the cold resistance of postharvest peach fruits [49]. KEGG enrichment analysis indicated that the genes displaying differential methylation were enriched primarily in pathways such as tryptophan metabolism, exopolysaccharide biosynthesis, monoterpenoid biosynthesis, and the biosynthesis of plant hormones. These findings suggest that methylation may play a role, either directly or indirectly, in regulating these processes.

DNA methylation levels are typically inversely correlated with gene transcription activity. Therefore, the extent of DNA methylation directly impacts gene expression levels, thereby influencing their functionality [50]. An analysis of the methylome map of apples revealed insights into the genome-wide relationship between DNA methylation and gene expression. Compared with genes with methylated promoters, genes with unmethylated promoters presented higher expression levels, suggesting a negative correlation between gene methylation and expression [51]. When watermelon seedlings were subjected to a 12 h treatment at 4 °C, there was a notable decrease in methylation rates in both diploid and triploid watermelon plants. In tomato fruits, cold treatment leads to the downregulation of the DNA demethylase *DML2*, resulting in hypermethylation of the promoter region. This hypermethylation silences genes associated with flavor volatile biosynthesis [50]. DNA methylation plays a role in plant stress responses by modulating the expression of relevant genes. In this study, eight candidate genes related to high-temperature tolerance were identified using WGCNA, and their expression levels were subsequently correlated with water methylation levels. The expression levels of five genes were positively correlated with the methylation level, whereas the expression levels of three genes were negatively correlated with the methylation level. This study suggests that DNA methylation in *G. hirsutum* can either increase or suppress gene expression transcriptionally under high-temperature stress conditions. Methylation that enhances gene expression is located primarily in the downstream or gene region, while methylation that suppresses gene expression is found mainly in the upstream region of the gene. Future investigations can explore the molecular regulatory mechanisms of high-temperature DNA methylation in cotton at the molecular and cellular levels. By exploring these mechanisms at the gene level, researchers can potentially advance the understanding of high-temperature stress responses in cotton and contribute to the development of new high-temperature-resistant cotton varieties.

## 5. Conclusions

In summary, this study conducted WGBS on the heat-resistant material Xinluzao 36 and the heat-sensitive material Che 61–72 at 0 h and 12 h of high-temperature stress treatment. High-temperature stress treatment increased the methylation level of CHH, and there were significant differences between the A and D subgenomes. The methylation density was inversely related to the distribution density of genes. Combined analysis with RNA-seq revealed that DNA methylation did not affect the expression of many genes (accounting for 85.68% of the differentially methylated genes). Our study highlights the importance of epigenetic modifications in cotton heat tolerance, which is highly important for future crop improvement strategies and technologies that consider epigenetic modifications. In addition, pathways such as the biosynthesis of plant hormones, thiamine metabolism, glutathione metabolism, and tyrosine metabolism may involve methylation variation, which requires further experimental verification.

## Data Availability

The RNA-seq data presented in this study have been deposited in the NCBI repository under accession number PRJNA706603. The WGBS data presented in this study have been deposited in the NCBI repository under accession number PRJNA1127292.

## Supplementary Materials

The following supporting information can be downloaded at https://www.mdpi.com/article/10.3390/genes15101241/s1, Table S1. Summary statistics for WGBS analysis of high-temperature stress treated samples from G. hirsutum. Figure S1. Coexpression network for the WGCNA of RNA-seq data.
