# Supplementary figures and images for: Whole-Genome Bisulfite Sequencing (WGBS) Analysis of Gossypium hirsutum under High-Temperature Stress Conditions
Source: Genes (Basel). 2024 Sep 24;15(10):1241. doi: 10.3390/genes15101241 (PMC11507439; doi:10.3390/genes15101241)

a

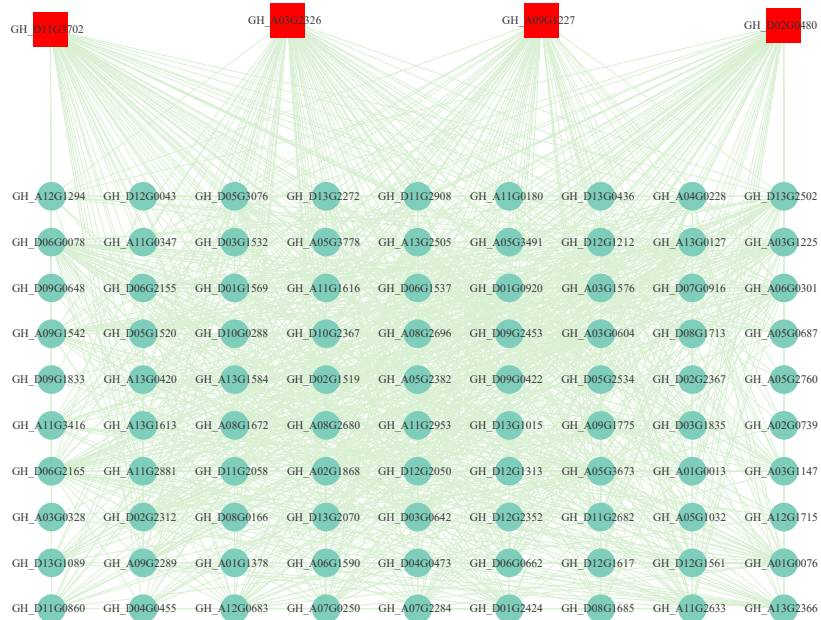

b

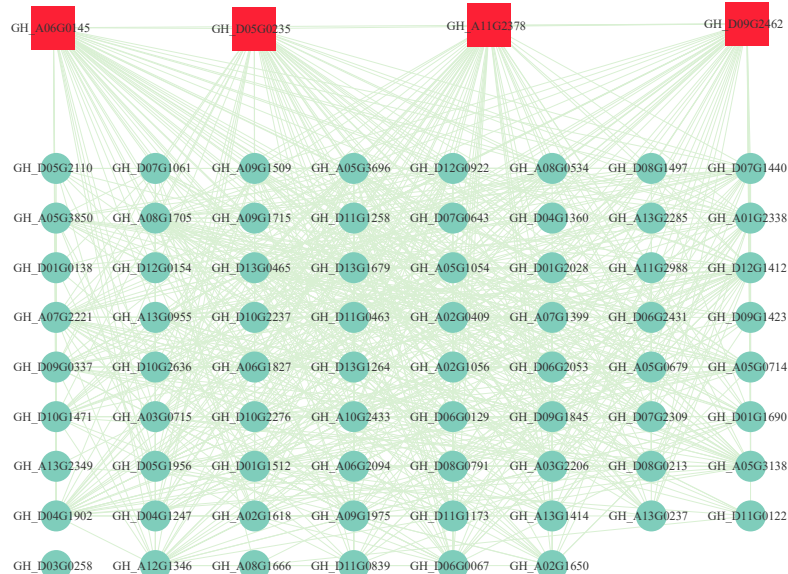

Supplement: Supplementary file 1 [file genes-15-01241-s001.zip › Figure S1.pdf]
